# Enantioselective Toxicity of Tetramethrin to Different Developmental Stages of Zebrafish (*Danio rerio*)

**DOI:** 10.3390/toxics12020146

**Published:** 2024-02-13

**Authors:** Jiqin Feng, Xintong Xu, Wenfei Huang, Honghong Gong, Xiaohui Sun, Jinsong Liu, Chao Xu, Weiping Liu

**Affiliations:** 1 Zhejiang Environment Technology Co., Ltd., Hangzhou 310000, China; 2 College of Environment, Zhejiang University of Technology, Hangzhou 310032, China; 221122270224@zjut.edu.cn; 3 Eco-Environmental Science Research & Design Institute of Zhejiang Province, Hangzhou 310007, China; 4 Zhejiang Environmental Monitoring Center, Hangzhou 310012, China; 5 Key Laboratory of Pollution Exposure and Health Intervention of Zhejiang Province, Interdisciplinary Research Academy (IRA), Zhejiang Shuren University, Hangzhou 310015, China

**Keywords:** pyrethroid, enantioselectivity, endocrine, immunotoxicity, life stages

## Abstract

Chiral pesticides exhibit enantioselective differences in processes such as biological absorption, metabolism, and toxic effects. Organisms have different physiological characteristics at different developmental stages. Therefore, conducting enantiomeric toxicity studies at different developmental stages of organisms can help deepen the understanding of the ecological effects of chiral pesticides. This study focused on *trans*-tetramethrin (Tet) and investigated the enantioselectivity in bioconcentration, developmental toxicity, estrogenic effects, and immunotoxicity of Tet’s racemate ((±)-Tet) and its two enantiomers ((+)-Tet and (−)-Tet) in three developmental stages of zebrafish: embryos, yolk sac larvae, and juveniles. The results showed that Tet exhibited different enantioselectivity in lethal, bioconcentration, and teratogenic effects on zebrafish at different developmental stages. The LC_50_ value was (+)-Tet > (±)-Tet > (−)-Tet, with embryos being the most sensitive, followed by juveniles and yolk sac larvae. The enantioselective bioconcentration was (±)-Tet > (+)-Tet > (−)-Tet, and the bioconcentration effect was greater in embryos than that in yolk sac larvae and juveniles. Developmental toxicity indicated that (+)-Tet and (±)-Tet had higher teratogenic effects on yolk sac larvae than on embryos. Tet exhibited different enantioselective effects on the expression of zebrafish estrogen-related genes and innate immune-related genes at different developmental stages. These results will contribute to a more comprehensive assessment of the aquatic toxicity and environmental risks of chiral pesticides.

## 1. Introduction

The sensitivity of organisms to pollutants varies during different stages of growth, especially during the early developmental stages when rapid cell differentiation and system development occur, accompanied by significant morphological and physiological changes [1]. Therefore, early exposure to toxins may permanently affect the normal development of organisms and even lead to death. For example, during the early developmental stages of zebrafish embryos, yolk sacs, and juvenal zebrafish, the detoxification organs have not yet formed, and they mainly rely on a layer of chorionic villi as a barrier to resist external pollutants [2]. However, during the yolk sac stage (48–120 hpf), the chorionic villi fall off and they rely entirely on their own nutrients to survive, at which point their detoxification organs have formed and grow rapidly [3]. In comparison, during the juvenal stage (from 120 hpf to sexual maturation), they can absorb drugs from the surrounding environment through multiple pathways such as the intestines, skin, and gills, and their gonads begin to differentiate [1]. In addition, pollutants can also interfere with the endocrine or immune systems of early developing zebrafish, causing significant changes in physiological indicators and morphology [4]. However, current environmental toxicology research mainly focuses on a particular life stage of the organism and cannot fully reflect the toxicity of pollutants.

Pesticides are extensively used in agricultural production and public health pest control, but they also pose significant ecological and environmental risks, making them a widespread type of pollutant in the environment [5]. Currently, at least 40% of commonly used pesticides have a chiral structure, and most are still sold and used in racemic form [6]. However, enantiomers contained in chiral pesticides have significant differences in biological effects such as enrichment, metabolism, lethality, teratogenicity, and endocrine disruption [7]. Synthetic pyrethroids (SPs) are a typical type of chiral pesticide, being one of the most widely used, efficient, and low-toxicity insecticides on the market, with a top three market share [8]. However, with the widespread use of SPs, their ecological risks and environmental impacts have increasingly attracted attention [9]. In particular, regarding their significant aquatic toxicity, in recent years, many scholars have conducted research and reported on the environmental residues, toxic effects, and toxicological mechanisms of SPs. For example, Delgado-Moreno et al. [10] detected SPs as high as 13,000 μg/L in a river in the United States. Magnuson et al. [11] found that SPs accumulate in aquatic organisms, causing lethal, teratogenic, hatching inhibition, gene expression interference, and other toxic effects on fish, thus disrupting the structure and function of aquatic ecosystems. Many SPs have been identified as actual or potential endocrine disruptors (EDCs), interfering with the development, reproduction, and immune function of organisms, affecting key gene expressions such as *cyp19a*, *cyp19b*, *vtg1*, and *vtg2* in the endocrine system, and *il-1β*, *1l-8*, *cxcl-cl*, and *cc-chem* in the immune system. For instance, Yang et al. [12] found that enantiomers of cypermethrin could induce the transcription of estrogen-related genes *vtg1* and *vtg2* in the liver of male zebrafish, exhibiting significant enantioselectivity. Park et al. [13] found that deltamethrin could increase the expression of inflammatory genes such as *1l-8* in zebrafish.

Tetramethrin (Tet) is one of the most effective synthetic pyrethroids (SPs) in the control of mosquitoes and flies, with rapid knockdown effects on such sanitary pests [14]. Tet has two chiral centers resulting in four stereoisomers, with its primary active components being the two enantiomers of *trans*-Tet. We hypothesized that the two enantiomers of *trans*-Tet might have different toxic effects and enantiomeric differences on zebrafish at various life stages. Thus, this study targets the enantiomers of the main active ingredient in Tet, *trans*-Tet, and its two enantiomers. Zebrafish at different developmental stages (embryo, yolk sac, and larvae) were selected to investigate their bioconcentration, acute toxicity, developmental toxicity, endocrine disruption effects based on *cyp19a*, *cyp19b*, *vtg1*, and *vtg2* genes, as well as immunotoxicity based on gene expression of interleukin-1β (*il-1β*), *1l-8*, *cxcl-clc*, *cc-chem*, etc. [15]. Considering the enantioselective environmental fate and toxicity of SPs, evaluating the enantioselective toxicity at different life stages can provide a more comprehensive basis for the risk assessment of chiral pesticides, which is crucial for the development of efficient and low-risk pesticides. Through exploring the effects and mechanisms of *trans*-Tet and its enantiomers on zebrafish, we aim to provide a more comprehensive toxicological reference for the scientific use and environmental safety assessment of SPs.

## 2. Materials and Methods

### 2.1. Chemicals and Enantiomer Preparation

Tet (98% purity, enantiomeric ratio: 1/9) was purchased from Sigma-Aldrich–Fluka (Bornem, Belgium). *n*-Hexane, ethanol, and acetone were all chromatography grade and purchased from Sigma-Aldrich (St. Louis, MO, USA). All other chemicals and solvents were of analytical grade. Enantiomer separation and preparation of Tet were performed using a chiral high-performance liquid chromatography (HPLC) system (Jasco, Tokyo, Japan) equipped with a PU-2080 pump, UV-2075 plus UV/visible detector, and a Chiralpak AD column (Tokyo, Japan). The mobile phase was *n*-hexane/ethanol (*v*/*v* = 95/5), with an injection volume of 20 μL and UV detection at a wavelength of 235 nm.

### 2.2. Zebrafish Maintenance and Embryo Collection

Adult zebrafish of the AB strain, aged 3–4 months, were obtained from the Institute of Hydrobiology of the Chinese Academy of Sciences in Wuhan, China. The zebrafish were kept in a controlled environment with a 12 h light/12 h dark cycle, a temperature of 28 ± 0.5 °C, water hardness of 45–63 mg/L, and pH of 7.2. They were fed twice a day with fairy shrimp, and red worms were added to the diet to obtain sexually mature zebrafish for spawning. A 2:1 male to female ratio was selected for spawning. The zebrafish were stimulated with light the next morning to induce egg laying. Newly fertilized eggs were collected within 1–2 h post fertilization (hpf) using a siphon. The eggs were washed thoroughly with sterilized and dechlorinated tap water three times, then soaked in a 1% methylene blue solution for over 30 min in a constant temperature water bath (28 °C). Afterwards, the eggs were rinsed with sterilized and dechlorinated tap water and placed in a 28 °C water bath. Dead eggs were removed immediately upon observation.

### 2.3. Exposure Experiments

Methods for acute toxic exposure reference OECD standards [16]. Briefly, 40 embryos (6 hpf), 40 larvae at the yolk sac stage (48 hpf), and 20 juvenile fish (60 days) were exposed to different concentrations (0.2, 0.5, 0.8, and 1 mg/L) of (±)-Tet, (−)-Tet, or (+)-Tet for 96 h. Every 24 h the development of zebrafish embryos and yolk sac stages was observed under an inverted dissecting microscope (Leica Microsystems, Wetzlar, Germany), recording mortality, heartbeat, and malformations. The content of dimethyl sulfoxide (DMSO) in the exposure solution was 0.2‰ (*v*/*v*), and all exposure solutions were prepared in 10% Hank’s solution, with the control group containing 0.2‰ DMSO in 10% Hank’s solution. Each exposure concentration had three parallels, and the exposure solution was changed every 24 h. During the exposure, zebrafish at each stage were placed in an artificial climate chamber with a temperature of (28 ± 1) °C and a light cycle of 14 h:10 h.

Based on the results of acute toxicity experiments, 1/10 and 1/100 of the 96 h lethal concentration (LC_50_) at each stage were taken as different subacute exposure concentrations of Tet for different stages of zebrafish [17] and were 5, 50 μg/L for the embryo stage, 10, 100 μg/L for the yolk sac stage, and 6.5, 65 μg/L for the juvenile stage, with exposure lasting 6 days. After exposure, 40 embryos, 40 yolk sacs, and 5 juvenile fish were taken to analyze the bioconcentration, represented by the bioconcentration factors (BCFs) using the formula BCF = C_FS_/C_WS_, where C_FS_ is the Tet content in the fish and C_WS_ is the Tet concentration in the water. Additionally, the effects of gene expression were analyzed using 25 embryos, 25 yolk sac stages, and 5 juvenile fish in each exposure concentration, with three parallels.

### 2.4. Determination of Tet Content in Exposure Solution and Juvenile Fish

The concentration of Tet in water samples was determined according to the previous method [18], that is, 5 mL of exposure solution at the beginning (T_0_) and before changing the solution (T_24_) was collected for each exposure group. The water samples were mixed with internal standard deltamethrin, then, 1 mL of *n*-hexane/ethyl acetate (1/1, *v*/*v*) was added for extraction and repeated twice. The combined organic layer was dried with nitrogen and dissolved in 0.1 mL of *n*-hexane for analysis. Three replicates were performed for each group.

For biological samples (n = 3), internal standard deltamethrin was added and then sonicated. After that, 1 mL of *n*-hexane/ethyl acetate mixture was used for extraction. The extract was transferred to a 5 mL Teflon centrifuge tube. After thorough mixing with 2 mL of ultrapure water and centrifugation at 6000 rpm for 2 min, the upper organic layer was collected. The extraction was repeated twice, and the combined organic layer was dried with nitrogen, dissolved in 0.5 mL of acetonitrile, and then mechanically shaken with 0.5 mL of acetonitrile-saturated petroleum ether for lipid removal. Lipid removal was repeated twice by removing the upper layer of the petroleum ether. The lower organic phase was dried with nitrogen, dissolved in 1 mL of (1/9, *v*/*v*) acetone/*n*-hexane, and further purified using a 500 mg Florisil SPE column (ANPEL Scientific Instrument Co., Shanghai, China). Elution was performed with 3 mL of (1/9) (*v*/*v*) acetone/*n*-hexane, followed by nitrogen drying and re-dissolving in 0.1 mL of *n*-hexane for GC-ECD analysis.

Sample analysis was performed using an Agilent GC 7890A system (Agilent Corporation, Santa Clara, CA, USA) equipped with an HP-5 capillary column (30 m × 0.25 mm i.d. × 0.25 μm) and an Electron Capture Detector (ECD). The operating conditions were as follows: initial temperature 200 °C (2 min), ramped at 6 °C/min to 270 °C, held for 10 min, injector temperature 220 °C, carrier gas high-purity N_2_, flow rate 1 mL/min, and detector temperature 300 °C.

### 2.5. Quality Assurance/Quality Control (QA/QC)

A standard curve with 7 concentration gradients (1, 2, 5, 10, 20, 50, and 100 μg/L) is prepared (R^2^ = 0.9980). The instrument is checked with the standard curve at the middle concentration every 12 h, and recalibration is required when the relative deviation of the response value of the calibrated compound is greater than 20%. The instrument blank is tested with blank *n*-hexane reagent every ten samples. The recovery experiment results show that the average recovery rates of Tet in water and biological samples are 98.2% and 76.4%, respectively, and the method detection limits for water and biological samples are 0.5 ng/mL and 3 ng/g, respectively.

### 2.6. RNA Extraction, Reverse Transcription, and Realtime PCR

The total RNA in the 20 exposed zebrafish embryos/yolk-stage larvae or 40 mg of juvenal liver tissue was extracted using Trizol reagent (Takara, Dalian, China), and then the RNA concentration was determined according to the instructions of the kit [19]. Based on the results of the calculations of the stability M of *β-actin*, *rpl8*, and *18sRNA*, it was decided that *18sRNA* would be the housekeeping gene. The SYBR Green Real-time PCR Master Mix (TOYOBO, Osaka, Japan) kit was used for a real-time quantitative PCR reaction on a 96-well PCR plate in a PCR instrument. The RNA obtained was reverse transcribed into cDNA using a kit from Kangwei Century (Beijing, China). The cDNA was uniformly diluted to a concentration of 500 ng/mL based on pre-experimental results. The PCR reaction program was set as follows: pre-denaturation at 95 °C for 1 min, followed by 40 cycles of denaturation at 95 °C for 15 s, and annealing/extension at 60 °C for 1 min. The formula for calculating the relative gene expression level is 2^−ΔΔCT^, where ΔCt = Ct(Target) − Ct(Ggapdh) [20]. The RNA samples were assessed by measuring the optical density (OD_260_/OD_280_) ratio using an ultramicro UV–Vis spectrophotometer (Bio TekeND5000, Beijing, China).

### 2.7. Statistical Analysis

The normality and homogeneity of the embryonic fish toxicity test data were assessed using the Kolmogorov–Smirnov and Levene methods, respectively. The data were expressed as mean ± standard deviation (SD), and a one-way analysis of variance (ANOVA) was used to evaluate the significance difference between the experimental and control groups, followed by Dunnett’s test. A two-tailed *t*-test was conducted to calculate the significant difference between the enantiomers. The symbols * and ** denote significant differences at *p* < 0.05 and *p* < 0.01, respectively. All statistical analyses were performed using SPSS 19.0 (SPSS Inc., Chicago, IL, USA).

## 3. Results

### 3.1. Enantiomeric Separation

**Figure 1:**
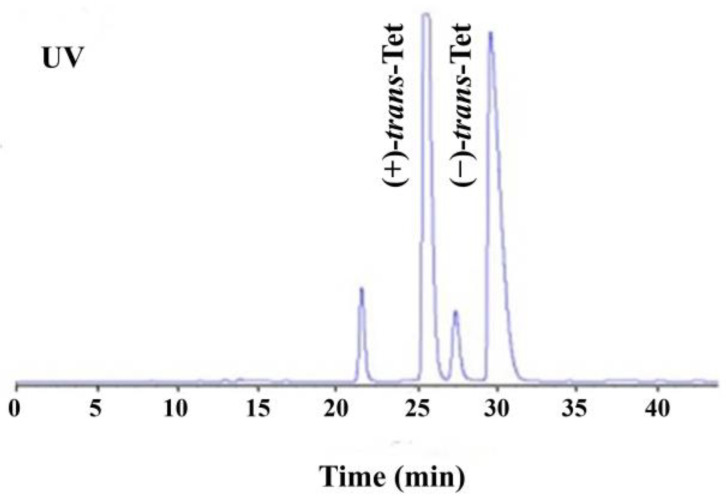
HPLC chromatogram of enantiomeric separation of Tet (UV: ultraviolet detector).

As shown in Figure 1, the four isomers of Tet were successfully baseline separated. Due to the 1:9 ratio of its *cis*/*trans* isomers and the fact that its activity is mainly determined by the *trans* isomers, the study focused on the racemic *trans*-Tet, as well as (±)-*trans*-Tet and its two enantiomers, (+)-*trans*-Tet and (−)-*trans*-Tet. The enantiomers were collected separately at the HPLC outlet, dried under nitrogen, redissolved in *n*-hexane, and stored at −18 °C in the dark. The determination of enantiomeric purity followed the above-mentioned enantiomer separation method. The results indicate that the purity of the prepared (+)-Tet and (−)-Tet is 97.4% and 96.9%, respectively.

### 3.2. Acute Toxicity

Table 1 shows the 48 h and 96 h LC_50_ values of zebrafish at different stages. Overall, the sensitivity sequence of zebrafish at different developmental stages to Tet is embryo m > juvenal > yolk sac. Toxicity increases with exposure time. The 96 h LC_50_ indicates that the toxicity sequence of enantiomers is (+)-Tet ((0.49 ± 0.53) mg/L) > (±)-Tet ((0.57 ± 0.66) mg/L) > (−)-Tet (>1 mg/L). In the embryo stage, exposure to high concentrations of (+)-Tet for 96 h resulted in a mortality rate of 87.5%, which is 1.4 and 6.4 times higher than that of the same concentration of (±)-Tet and (−)-Tet, respectively. In the juvenal stage, the mortality rate of the high-concentration group of (+)-*trans*-Tet for 96 h was 61.3%, which is 1.1 and 6.2 times higher than that of (±)-Tet and (−)-Tet, respectively.

### 3.3. Bioconcentration

**Figure 2:**
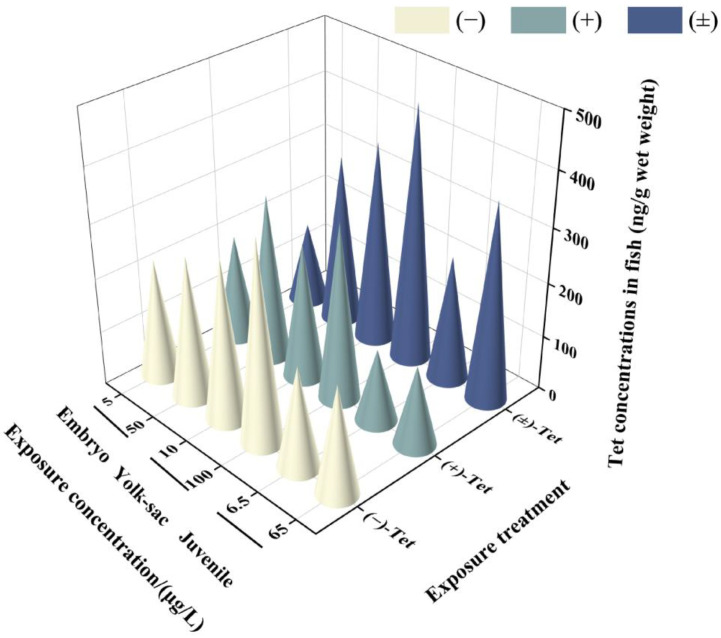
Concentrations of Tet and its enantiomers in zebrafish at different stages after 6 days of exposure.

Analysis of the Tet concentrations in each exposure group revealed that the actual initial concentration (T_0_) in each group was 76.5–89.6% of the nominal concentration, and the actual concentration before renewal (T_24_) decreased to 28.6–59.5% of the nominal concentration. This decrease may be attributed to the strong hydrophobicity of the pyrethroid, which makes it prone to adsorption on the walls of the exposure containers. However, these concentrations still meet the requirements for calculating BCF by OECD, as the actual concentration of the exposure solution should be maintained at 20% or more of the initial value [17]. No isomerization between enantiomers was observed in the solution during the exposure period. The water concentration used for BCF calculation was the measured concentration at T_24_. The results indicate that after 6 days of exposure, Tet showed different bioconcentration effects in zebrafish at different developmental stages (Figure 2). The lowest enrichment was observed in the low-concentration exposure group of juvenal fish (146.89 ± 29.51) ng/g, while the highest was observed in the high-concentration exposure group of yolk sac larvae (469.59 ± 2.52) ng/g. In all exposure groups at each stage, the BCF value of (+)-Tet was greater than the BCF value of (−)-Tet (Table 2), consistent with the result that the acute toxicity of (+)-Tet is greater than that of (−)-Tet, indicating that the difference in acute toxicity between the Tet enantiomers may be due to their differential enrichment ability in zebrafish. Except for the low-concentration exposure group of embryos, the BCF of (±)-Tet in all exposure groups was greater than the BCF value of the individual enantiomers, suggesting a potential synergistic effect between the enantiomers in the process of biological enrichment. Although the BCF at the embryo stage was greater than the BCF of the yolk sac and juvenal fish, there was no significant difference in BCF values at each stage.

### 3.4. Developmental Toxicity

**Figure 3:**
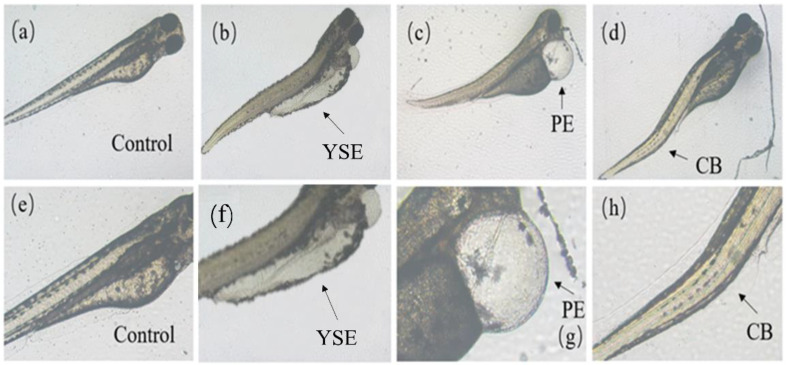
Malformations caused by Tet and its enantiomers at different developmental stages of zebrafish. (**a**–**d**) are control, yolk sac edema (YSE), pericardial edema (PE), and curved body (CB), respectively. The area of malformation is indicated by the black arrow. (**e**–**h**) are the enlarged view of (**a**–**d**), respectively.

As shown in Figure 3, zebrafish at the embryo and yolk sac stages exposed to Tet and its enantiomers for 48 h exhibited a variety of developmental abnormalities, including yolk sac edema (YSE), pericardial edema (PE), and curved body (CB). Based on the frequency and intensity of these abnormalities, PE and YSE were selected for statistical analysis of the malformation rate.

**Figure 4:**
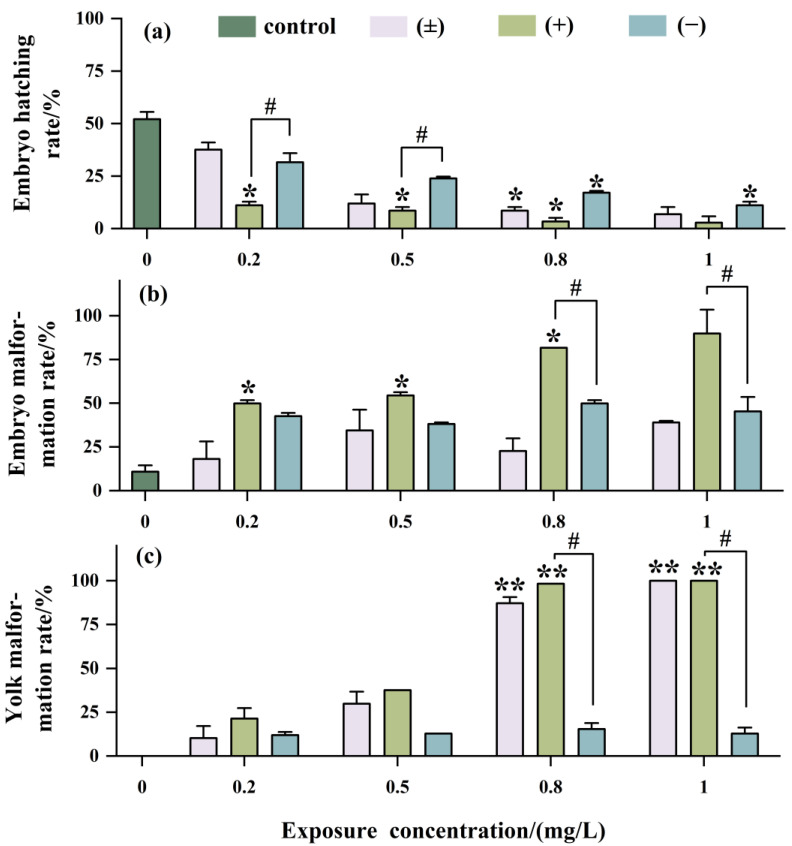
Developmental toxicity after being exposed to Tet and its *trans*-enantiomer for 48 h. Hatching rate (**a**), malformation rate (**b**), and yolk malformation rate (**c**). The symbol * denotes *p* < 0.05, while ** denotes *p* < 0.01 in comparison to the control. # indicates that there is a significant difference between (+) and (−)-Tet exposure groups at the same concentration (*p* < 0.05).

As shown in Figure 4a, with increasing exposure concentration, the hatching rate of embryos significantly decreased, with the inhibition of embryo hatching being most significant with (+)-Tet, reaching as low as 2.5% at an exposure concentration of 1 mg/L. The teratogenicity of (+)-Tet was 2.25 times and 2 times that of (±)-Tet and (−)-Tet, respectively (Figure 4b). There was no significant difference between (−)-Tet and (±)-Tet, which may be due to a certain antagonistic effect between different enantiomers.

The developmental toxicity at the yolk sac stage exhibited a concentration-dependent effect similar to that observed at the embryo stage (Figure 4c). However, the developmental toxicity in the (±)-Tet exposure group at the yolk sac stage was increased significantly with increasing exposure concentration. Enantiomeric selectivity analysis showed that at a concentration of 1 mg/L, the pericardial edema rate in the (±)-Tet and (+)-Tet exposure groups at the yolk sac stage reached 100%, while the pericardial edema rate in the (−)-Tet group was only about 15%.

### 3.5. mRNA Expression Levels of cyp19 and vtg Genes

**Figure 5:**
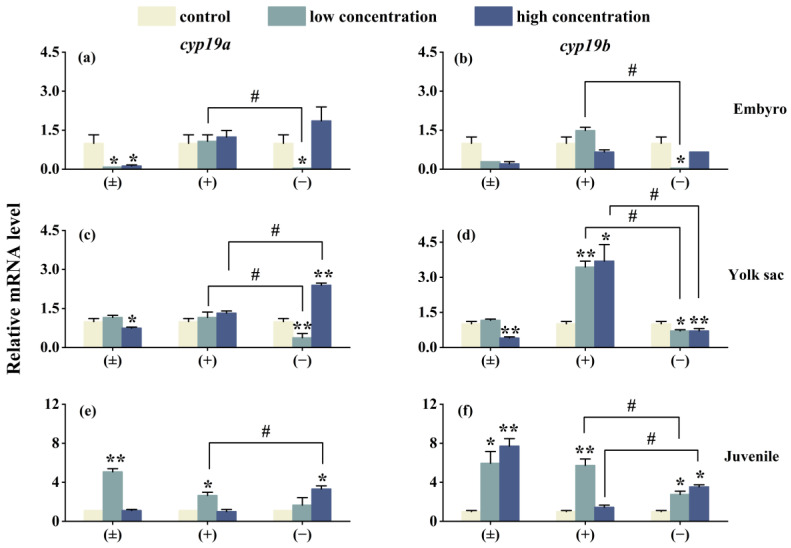
The expression of *cyp19a* and *cyp19b* in zebrafish embryo (**a**,**b**), yolk sac (**c**,**d**), and juvenal fish (**e**,**f**) after 6 days of exposure to Tet and its enantiomers. The symbol * denotes *p* < 0.05, while ** denotes *p* < 0.01 in comparison to the control. # indicates that there is a significant difference between (+) and (−)-Tet exposure groups at the same concentration (*p* < 0.05).

As shown in Figure 5, after 6 days of exposure, the expression of the genes *cyp19a*, *cyp19b*, *vtg1*, and *vtg2* in three different life stages of zebrafish is affected to varying degrees. In the high-concentration (±)-Tet exposure group, the expression of *cyp19a* and *cyp19b* genes in the embryo and yolk sac stages is suppressed, while an upregulation is observed in the juvenile stage. (+)-Tet exposure has an upregulating effect on the *cyp19a* and *cyp19b* genes in the three different life stages of zebrafish, especially in the yolk sac low- and high-concentration groups, as well as the juvenile low-concentration group. The low-concentration (−)-Tet significantly inhibits the expression of genes in the embryo and yolk sac stages, but upregulates the expression of these two genes in the juvenile stage. Significant enantioselectivity is observed in the exposure groups at each stage, with the gene expression of *cyp19a* and *cyp19b* in the embryo low-concentration (+)-Tet exposure group being 52 and 85.9 times that of the (−)-Tet group, respectively.

**Figure 6:**
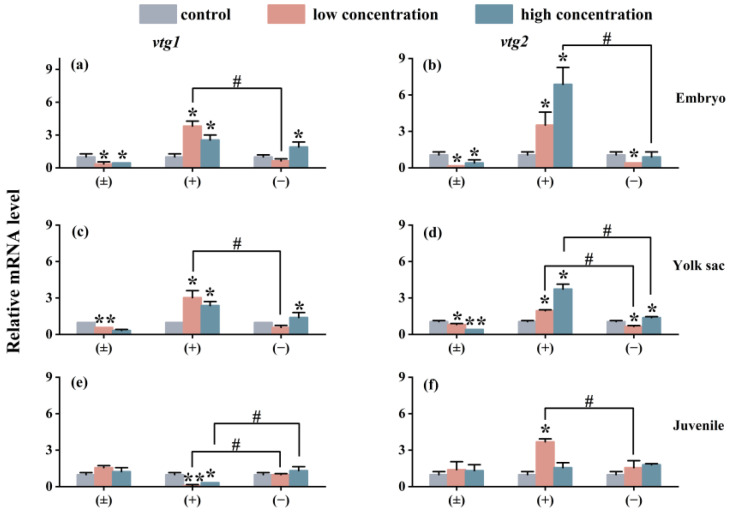
The *vtg1* and *vtg2* expression of zebrafish embryo (**a**,**b**), yolk sac (**c**,**d**), and juvenal fish (**e**,**f**) after 6 days of exposure to Tet and its enantiomers. The symbol * denotes *p* < 0.05, while ** denotes *p* < 0.01 in comparison to the control. # indicates that there is a significant difference between (+) and (−)-Tet exposure groups at the same concentration (*p* < 0.05).

The impact of exposure on the expression of the *Vtg* gene in each exposure group is similar to the situation of the *cyp* gene. As shown in Figure 6, compared with the control group, the (±)-Tet exposure group significantly inhibited the expression of the *vtg* gene in the embryo and yolk sac stages, while the (+)-Tet exposure group significantly upregulated the expression of the two genes. At the juvenal stage, *vtg1* was significantly downregulated in both the low- and high-concentration groups of (+)-Tet, which was significantly different from the other two stages. Therefore, significant enantiomeric selective differences can be observed in the exposure groups at each stage. In the low-concentration exposure group during the embryo stage, the gene expression of *vtg1* and *vtg2* in the (+)-Tet group was 6.1 and 11.2 times that of the (−)-Tet group, respectively.

### 3.6. Impact on the Transcription of Innate Immune-Related Genes

**Figure 7:**
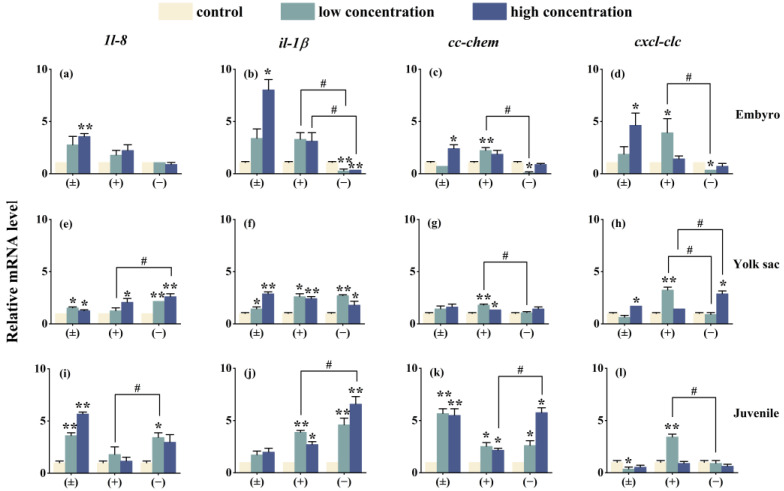
The expression of innate immune-related cytokines of zebrafish embryo (**a**–**d**), yolk sac (**e**–**h**), and juvenal fish (**i**–**l**) after exposure to Tet and its enantiomers for 6 days. The symbol * denotes *p* < 0.05, while ** denotes *p* < 0.01 in comparison to the control. # indicates that there is a significant difference between (+) and (−)-Tet exposure groups at the same concentration (*p* < 0.05).

After exposure to Tet enantiomers for 6 days, the expression of immune system-related genes is shown in Figure 7. Except for the *cxcl-clc* gene in the embryo stage (−)-Tet group and the juvenile stage (±)-Tet group, the expression of most immune genes in zebrafish at each stage was significantly upregulated compared to the control group. Significant enantioselective immune toxicity was observed in all three developmental stages. For example, in the embryo stage under low-concentration exposure conditions, the gene expression of *il-1β*, *cc-chem*, and *cxcl-clc* in the (+)-Tet group was 13.6, 29.1, and 13.1 times higher, respectively, compared to the (−)-Tet group.

## 4. Discussion

Residues of SPs in water pose a significant risk to aquatic life [21], and the chiral nature of SPs, along with the varying sensitivities at different life stages of organisms, adds complexity to this issue. In our study, Tet and its enantiomers exhibited distinct enantioselective toxicities in zebrafish across three developmental stages. The 96 h-LC_50_ values revealed notable differences in the acute toxicity of Tet and its enantiomers in zebrafish, with the toxicity sequence being (+)-Tet > (±)-Tet > (−)-Tet. This difference may be attributed to the enantioselective bioconcentration of Tet in zebrafish, which, according to our experiments, followed the order of (±)-Tet > (+)-Tet > (−)-Tet. Despite the racemate having a higher bioconcentration capacity than (+)-Tet, its acute toxicity was lower, suggesting an antagonistic interaction between (−)-Tet and (+)-Tet. Zebrafish at various developmental stages showed differences in sensitivity to the same contaminant, with embryos being more susceptible than juveniles and yolk sac stages, likely due to increased bioconcentration of Tet during the embryonic stage. Furthermore, previous research indicates that Tet impairs the oxygen uptake capacity of juvenile gills [22], which could also explain the heightened sensitivity of juveniles compared to the yolk sac stage.

Bioconcentration is a crucial factor influencing the toxicity of pollutants [23]. In this study, the highest bioconcentration of Tet in zebrafish was recorded at 469.59 ng/g, surpassing previous findings on the bioconcentration of SPs in marine mammals like dolphins (68.4–7.04 ng/g) reported by Mariana B et al. [24]. In contrast, Corcellas et al. [25] observed the highest residual SP level of 4938 ng/g (ww) in wild river fish. This discrepancy could be attributed to dolphins being mammals equipped with enzymes to metabolize SPs, whereas fish either lack these enzymes or possess them in minimal amounts, leading to a greater accumulation of Tet in fish. The study also noted variations in the BCF values of zebrafish at different developmental stages, with the embryo stage showing a higher BCF value than the other stages. This could be due to the embryonic chorion’s adsorption of Tet and a comparatively lower metabolic capacity at this stage. Similar observations were made by Tu et al. [26], who reported that zebrafish embryos exhibited significantly higher enrichment kinetics for permethrin, deltamethrin, and lambda-cyhalothrin compared to their juvenile and adult stages. Moreover, within a 24 h depuration period, embryos could not fully eliminate SPs from their system. Furthermore, the BCF value for the (+)-Tet group was higher than that of the (−)-Tet exposure group, indicating a selective bioconcentration of Tet enantiomers in zebrafish [27], likely due to the selective absorption or metabolism of Tet by zebrafish.

Developmental toxicity is more sensitive than acute toxicity. In this study, hatching inhibition is a primary effect of Tet on zebrafish embryos, with a hatching rate of only 2.5% when exposed to 1 mg/L (+)-Tet. Similar findings were also observed in a previous study [28]. Moreover, both zebrafish embryos and yolk sac larvae exhibited various degrees of cardiac malformations (such as pericardial edema) following Tet exposure, with comparable phenomena noted with other SPs [29,30]. It is noteworthy that Tet shows enantioselective differences in toxicity to zebrafish at the same life stage, with (+)-Tet inducing malformations more effectively than (−)-Tet, aligning with the enantioselectivity of Tet in bioconcentration and acute toxicity. Additionally, our findings suggest that the malformation rate after exposure to (+)-Tet and (±)-Tet at the yolk sac stage is higher than at the embryo stage, whereas the malformation rate after exposure to (−)-Tet at the yolk sac stage is lower than at the embryo stage. This could be attributed to the development of the liver in zebrafish at the yolk sac stage, which enhances the selective metabolism of (−)-Tet. Furthermore, there might be an antagonistic effect between (+)-Tet and (−)-Tet at the yolk sac stage, leading to a stronger teratogenic effect of the racemate at this stage.

The enzyme cytochrome P450 aromatase CYP19 plays a crucial role in the metabolism and transformation of hormones in fish [31]. CYP19 exhibits several isoforms, with Cyp19a being essential for gonadal differentiation in bony fish. Vitellogenins (Vtgs) are typically produced in the liver in response to estrogen [32]. The expression of these genes is frequently monitored to identify endocrine-disrupting chemicals (EDCs). In this study, the impact of Tet on four genes, *cyp19a*, *cyp19b*, *vtg1*, and *vtg2*, was observed to be consistent. During the embryo and yolk sac stages, both (±)-Tet and (−)-Tet suppressed the expression of these genes, whereas (+)-Tet enhanced their expression. In the juvenile stage, with the exception of the *vtg1* gene in the high-concentration (+)-Tet exposure group, the expression of the other genes increased to various extents across all exposure groups. The expression levels of the *cyp19a*, *cyp19b*, *vtg1*, and *vtg2* genes are significantly associated with the estrogenic effect [33]. Moreover, in zebrafish, *vtg1* is the primary yolk protein precursor, constituting the bulk of its content, while *vtg2* not only serves as a yolk protein precursor but also aids in the embryos’ adaptation to specific environments [21]. In this study, the observed differences in endocrine gene interference across various developmental stages could be attributed to the undeveloped gonads of zebrafish in the initial two stages, where the involved enzymes or receptors are similar. However, in the juvenile fish stage, as their gonads begin to develop, the involved enzymes or receptors diverge from those in the earlier stages [34]. Consequently, Tet exposure may result in hormonal imbalances in zebrafish at different stages, leading to varying degrees of reproductive toxicity. Moreover, Tet and its enantiomers demonstrate variations in estrogenic disruptive effects. Similar disparities in enantioselectivity in estrogenic activity [35] have been noted in previous studies on other chiral pesticides, primarily due to the differential binding of enantiomers to estrogen receptors. Additionally, our findings reveal differences, or even completely opposite interference effects, between the low-concentration and high-concentration exposure groups. This suggests that different enantiomers exhibit distinct concentration–response relationships, potentially explaining the enantioselective differences observed.

The innate immune system serves as the sole defense against external threats like pathogen invasion during the early development stages of zebrafish [36]. Chemokines, a set of non-antibody proteins produced by immune cells, play crucial roles in regulating and mediating immune, inflammatory, and hematopoietic responses. To understand how the innate immune system reacts to Tet and its enantiomers, we analyzed the gene expression levels of *1l-8*, *il-1β*, *cc-chem*, and *cxcl-clc* at three different developmental stages of zebrafish. The findings reveal that, with the exception of four immune genes in the (−)-Tet group and the *cc-chem* gene in the (±)-Tet group during the embryonic phase, all other genes experienced varying degrees of upregulation. This pattern mirrors the interaction of Tet with four critical endocrine system genes in zebrafish across different stages, suggesting potential inflammatory effects post-exposure and highlighting the bidirectional relationship between the endocrine and immune systems [37]. Furthermore, it was noted that the expression of certain immune genes in the exposed groups did not follow a concentration-dependent manner. For instance, the expression of the *cxcl-clc* gene in the (+)-Tet-exposed group was significantly or extremely upregulated during low-concentration exposure across various zebrafish stages, but this upregulation diminished at higher concentrations. This could be attributed to the higher toxicity of (+)-Tet, where low-concentration exposure activates the zebrafish immune system, whereas higher concentrations can harm the immune system, leading to a reduced immune response.

Overall, our research demonstrates that Tet influences zebrafish at various developmental stages, affecting both growth and development as well as the expression of endocrine and innate-related genes. Additionally, there are notable enantioselective differences at each developmental stage. These findings suggest that performing enantioselective toxicity studies at different life stages of organisms is beneficial for a thorough understanding of the ecological impacts of chiral pesticides.

## Figures and Tables

**Table 1:** LC_50_ of Tet and its enantiomers to zebrafish at different life stages.

Life Stages	Toxic Endpoint	Exposure Time	LC_50_ (mg/L, Mean ± SD)		
			(±)	(+)	(−)
Embryo	Coagulated embryos or lack of heartbeat	48 h	0.77 ± 0.09	0.69 ± 0.53	>1
		96 h	0.57 ± 0.66	0.49 ± 0.53	>1
Yolk sac larvae	Lack of movement or heartbeat	48 h	>1	>1	>1
		96 h	>1	>1	>1
Juvenile	Death	48 h	0.94 ± 0.66	>1	>1
		96 h	0.75 ± 0.83	0.65 ± 0.61	>1

**Table 2:** BCF values of Tet and its enantiomers in zebrafish at various stages after 6 days of exposure.

Stage	Chemicals	Exposure Concentration (µg/L)	BCFs
Embryo	(±)-Tet	5.00	38.89
		50.00	14.38
	(+)-Tet	5.00	52.27
		50.00	5.74
	(−)-Tet	5.00	35.02
		50.00	6.37
Yolk sac	(±)-Tet	10.00	37.5
		100.00	7.28
	(+)-Tet	10.00	26.97
		100.00	3.84
	(−)-Tet	10.00	16.48
		100.00	3.43
Juvenile	(±)-Tet	6.50	45.23
		65.00	9.94
	(+)-Tet	6.50	32.88
		65.00	3.59
	(−)-Tet	6.50	13.53
		65.00	2.03

## Data Availability

The data that support the findings of this study are available upon reasonable request from the corresponding author.
